# Importance of the novel organic cation transporter 1 for tyrosine kinase inhibition by saracatinib in rheumatoid arthritis synovial fibroblasts

**DOI:** 10.1038/s41598-017-01438-4

**Published:** 2017-04-28

**Authors:** Saliha Harrach, Bayram Edemir, Christian Schmidt-Lauber, Thomas Pap, Jessica Bertrand, Giuliano Ciarimboli

**Affiliations:** 1 Institute of Experimental Musculoskeletal Medicine, University Hospital Muenster, Muenster, 48149 Germany; 2 Experimental Nephrology, Department of Internal Medicine D, University Hospital Muenster, Muenster, 48149 Germany; 3 Clinic and Polyclinic of Internal Medicine IV, Hematology and Oncology, University Hospital Halle, 06120 Halle (Saale), Germany; 4 Department of Orthopedic Surgery, Otto-von-Guericke University Magdeburg, 39120 Magdeburg, Germany

## Abstract

Recent therapeutic approaches of rheumatoid arthritis (RA) address the use of small molecules such as tyrosine kinase inhibitors (TKIs). However, the TKIs developed to date have important side effects and/or scarce efficacy in inflammatory diseases such as RA. Since intracellular effective TKIs must enter the cell to reach their intracellular targets, here we investigated the interaction of the TKI saracatinib, a dual inhibitor of c-Src and c-Abl signaling, with transporters for organic cations as well as the role of these transporters for the biological effect of saracatinib in human RA-synovial fibroblasts (hRASF). Saracatinib significantly reduced proliferation of hRASF. The cellular saracatinib uptake was mainly dependent on the human novel organic cation transporter 1 (hOCTN1), which showed the highest apparent affinity for saracatinib among all other transporters for organic cations analyzed here. In hRASF, saracatinib biologic function was dependent on hOCTN1. Further analysis showed that disease specific factors (pH, inflammatory cytokines such as TNFα) regulated saracatinib uptake in hRASF. The knowledge of which transporters mediate the specific uptake of TKIs in target cells and of how the expression and function of such transporters are regulated in RA is of highest priority to develop effective drugs for successful therapy with minimal side-effects.

## Introduction

Rheumatic diseases such as rheumatoid arthritis (RA) are chronic and debilitating inflammatory diseases, for which there is currently no cure, and which require long-term symptomatic treatment. RA causes progressive synovial inflammation and results in irreversible degradation of joints, particularly of the bone and cartilage, which ultimately leads to chronic disability and premature mortality^1^. Activated synovial fibroblasts are engaged in the initiation and perpetuation of RA^2^ and for this reason represent potential target cells in the RA therapy. Compared with normal synovial fibroblasts, RASFs show changes in morphology and behavior, alterations in signaling cascades, different apoptosis responses and expression of adhesion molecules as well as matrix-degrading enzymes^2^. Moreover, RASF resemble in many aspects cancer cells, acquiring a permanently aggressive, tumor-like phenotype that mediates cartilage destruction^3^.

The introduction of biologicals has improved the treatment possibilities for patients affected by RA^4^. However, biologicals are often cumbersome to administer, requiring injection or infusion, are very expensive, and, more importantly, a considerable proportion of patients do not respond to these drugs^5^.

An important characteristic of inflammatory diseases is the presence of an intense cytokine signaling with activation of several cellular protein kinases. In RA many signaling pathways regulating function and differentiation of inflammatory cells are activated by both receptor and non-receptor tyrosine kinases (TKs)^6^. Indeed, it has been found that proteins of the RA synovial tissue are extensively phosphorylated by intracellular TKs^7^. Therefore, there is a strong interest in TK inhibitors (TKIs) as “small molecules” for RA therapy^6, 8^. Such “small molecules” have a comparable risk versus benefit profile of currently available biologic agents combined with the advantage of low costs^9^ and of oral administration, which is of pivotal importance in determining patients’ compliance and hence treatment success^4^. However, to date clinical effects fell short of the expectations deriving from *in vitro* data.

TK dependent pathways activated in RA include the Janus kinases/signal transducers and activators of transcription (JAK/STAT) pathway, spleen tyrosine kinase (Syk), c-Src, focal adhesion kinase (FAK), and c-Abl signaling^6^. In this context, the TKI saracatinib is of special interest, because it acts as a dual kinase inhibitor, with selective actions as c-Src- and c-Abl-TKI^10^. Although saracatinib has been originally developed for oncologic indications, it is now recognized that the Src kinase family is involved in multiple biological processes across different organ systems and for this reason saracatinib has become of special interest for repositioning programs^11^. Src kinases have manifold influences on fibroblasts: they activate FAK, which is crucial for transmission of integrin signaling upon adhesion of fibroblasts to the extracellular matrix (ECM), and promotes differentiation from resting fibroblasts into myofibroblasts^12^, fibroblasts motility, cell attachment, and migration^13^. c-Src has also a predominant role in osteoclast formation and therefore bone resorption^14^. Src family kinases induce transphosphorylation of PDGF receptor (PDGFR) upon ligand binding^15^. In turn, PDGFR stimulation is well known to activate c-Abl^16^, which has also been a promising target in recent studies on RA^17^. Both PDGFR and its ligands are overexpressed in RA synovial tissue, and PDGF is a potent stimulant of synovial hyperplasia in RA^17^.

As already outlined above, TKIs are not yet fully accepted as RA therapeutics because of their side effects and/or scarce efficacy. It must be underlined that the development of TKI as drug has been exclusively based on their inhibitory potency on TK activity, neglecting the question of how TKI can reach their intracellular targets. Because TKI are orally administered, most of them are of hydrophilic nature. Hydrophilic drugs need specific transport systems to reach their intracellular targets. Even though it is well known that such membrane transporters are of critical importance in determining drug effects and side effects^18^, there is little knowledge on membrane transporter expression and regulation in rheumatic diseases such as RA. Most TKI are positively charged molecules at neutral (e.g. saracatinib, pacritinib, fedratinib, PRT062607) or acidic pH (e.g. baricitinib, ruxolitinib, PRT062070, tofacitinib) and for this reason belong to the class of organic cations (OCs). OCs cannot freely pass the cell membrane and need to be transported into the cell, where they exert their function. This fact allows the accomplishment of specific cell targeting, as the expression of these transporters is organ and tissue specific. OCs are substrates for transporters of organic cations. The main members of this group of polyspecific transporters are the organic cation transporters 1–3 (OCT1-3), the novel organic cation transporters 1 and 2 (OCTN1 and 2), and the multidrug and toxin extrusion transporters 1 (MATE1). There are some differences concerning the way these transporters work: OCT1-3 are Na^+^- and pH-independent, OCTN1 and MATE1 are pH-dependent and OCTN2 is Na^+^-dependent^19^. Overall, the physiological role of these transporters seems to consist in the regulation of the concentration of positively charged endogenous molecules such as serotonin, dopamine and histamine and/or of their metabolites.

In this work, we investigated the uptake pathways and effects of saracatinib in RA synovial fibroblasts (RASF) under RA relevant conditions. We identified the human OCTN1 (hOCTN1) as the transporter for saracatinib in RASF and studied its regulation under pro-inflammatory conditions. Moreover, we showed that saracatinib is able to inhibit the PDGF-induced proliferation of human RASF.

## Results

### Saracatinib transport is mediated by OCTs

**Figure 1:**
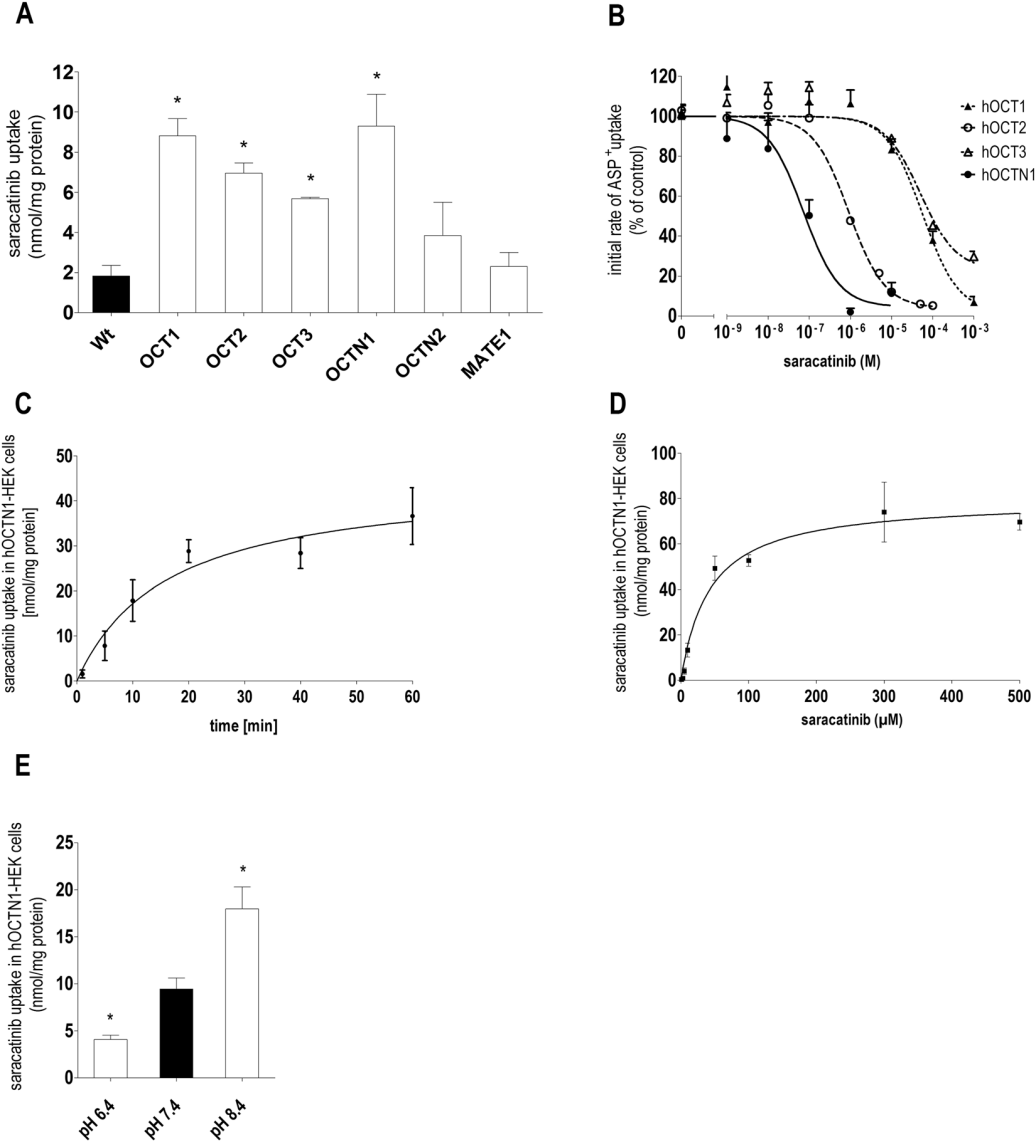
Characteristics of saracatinib uptake in HEK293 cells stably transfected with transporters of organic cations. (**A**) Specific saracatinib uptake by HEK293 cells overexpressing hOCT1-3, hOCTN1-2, and hMATE1 after 10 min incubation with saracatinib (10 µM) given as difference of accumulation at 4 °C and at 37 °C measured by the HPLC-UV method (n = 5–9). (**B**) Inhibition of initial ASP^+^ uptake in HEK293 cells overexpressing hOCT1 (▴), hOCT2 (⚬), hOCT3 (▵) or hOCTN1 (⦁) by increasing saracatinib concentrations. Values are mean ± SEM expressed as percentage of ASP^+^ uptake in the absence of saracatinib (n = 12–18). (**C**) Time-dependent saracatinib uptake by hOCTN1-HEK293 cells using a fixed concentration of 10 µM (n = 3). (**D**) Concentration-dependent uptake of saracatinib by hOCTN1- HEK293 cells (n = 12) after 10 min incubation, based on linearity of uptake determined from time-dependent saracatinib uptake studies (s. 1 C). (**E**) Influence of extracellular pH on saracatinib accumulation into hOCTN1-HEK293 cells (n = 12). Asterisks * indicate significant differences of saracatinib uptake in HEK293-WT cells (**A**) or at pH 7.4 (**D**), calculated by one-way ANOVA followed by Dunnett’s test (*P* < 0.05).

We analyzed the saracatinib uptake in HEK293 cells stably overexpressing the single human hOCTs using a HPLC method. hOCT1–3, and hOCTN1-overexpressing cells displayed a significantly increased saracatinib accumulation than WT-HEK293 cells (8.8 ± 0.9, 7.0 ± 0.5, 5.7 ± 0.1, and 9.3 ± 1.6 nmol/mg protein versus 1.8 ± 0.5 nmol/mg protein for hOCT1-3, hOCTN1-, and WT-HEK293 cells, respectively, Fig. 1A). In contrast, there was no significant difference in saracatinib uptake between both hOCTN2- or hMATE1-expressing cells (3.8 ± 1.7 and 2.3 ± 0.7 nmol/mg protein, respectively) in comparison to WT-HEK293 cells (Fig. 1A). The saracatinib uptake of control (empty vector transfected or non doxycycline induced) transfected HEK293-cells was not different from WT-HEK293 cells (not shown). The saracatinib uptake observed in control HEK293 cells is probably due to the low endogenous expression of transporters (OCT1-3 and OCTN1), which are able to mediate the transport of saracatinib (Supplementary Fig. 2). Next, we compared the apparent affinities (IC_50_) of the potential saracatinib transporters (hOCT1-3, and hOCTN1) by inhibiting the uptake of their model substrate ASP^+^ with saracatinib (Fig. 1B). Estimated IC_50_ values indicated a preferential interaction of saracatinib with OCTN1 (IC_50_ = 72 nM), whereas OCT1-3 (IC_50_ = 57, 0.9, and 50 µM for OCT1-3, respectively) revealed comparatively lower apparent affinities for saracatinib. The time course of hOCTN1 mediated saracatinib accumulation in HEK293 cells was found to be linear for at least 15 min (Fig. 1C). Thus, a 10 min incubation time was selected for further studies. Cellular accumulation kinetics revealed that saracatinib uptake by hOCTN1-HEK293 cells is a saturable process with a K_m_ of 42 ± 11 µM and a maximum saracatinib uptake (*V*_max_) of 79.7 ± 5.2 nmol/mg protein (Fig. 1D). According to its properties as H^+^/organic cation antiporter, hOCTN1 mediated saracatinib uptake was pH dependent (Fig. 1E).

### RASF mainly accumulate saracatinib via hOCTN1

**Figure 2:**
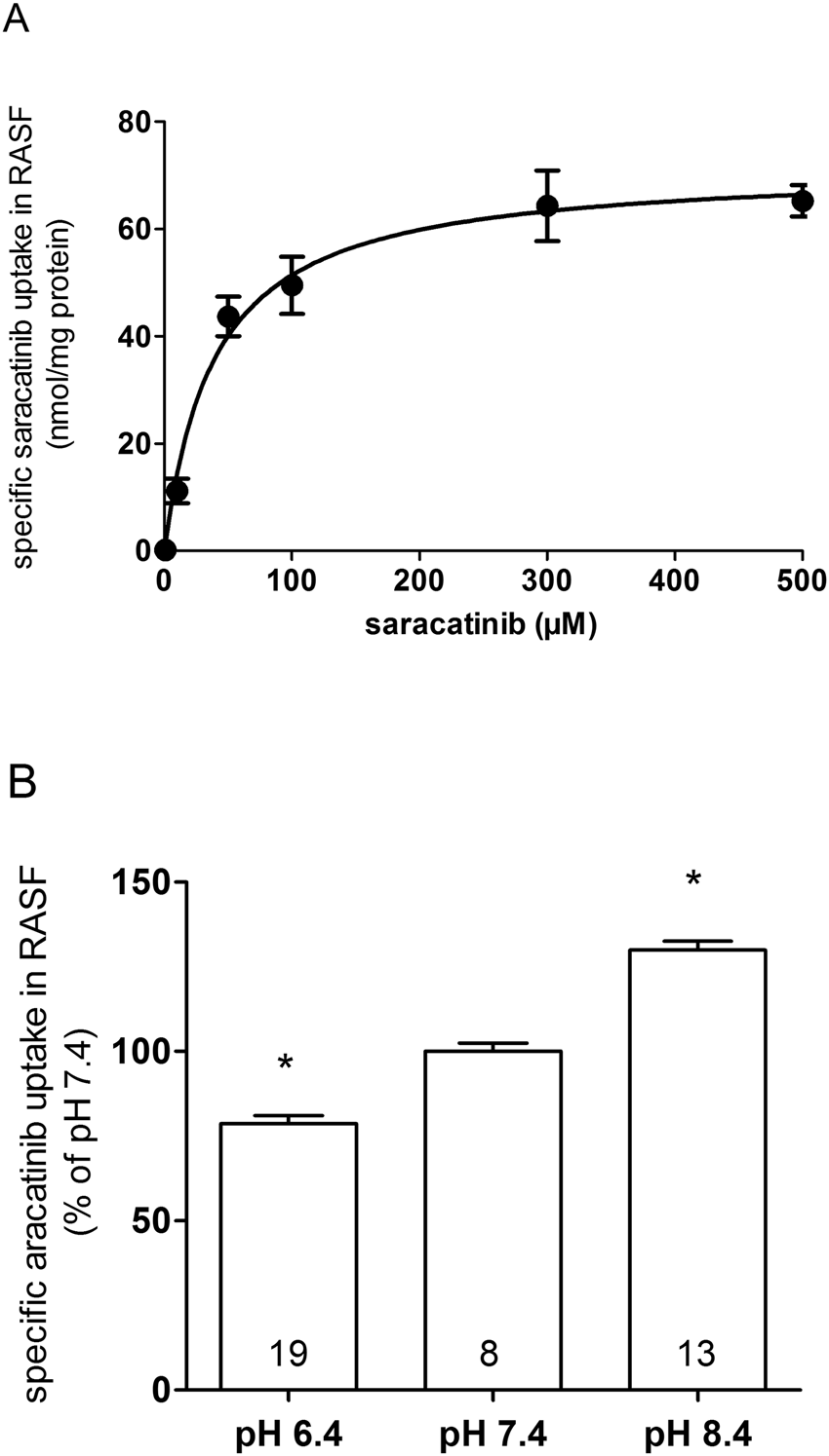
Characteristics of saracatinib uptake in hRASF. (**A**) Concentration-dependent uptake of saracatinib by RASF after 10 min incubation (n = 6–16) using the HPLC-UV method to quantify intracellular saracatinib levels. The kinetic parameters K_m_ and *V*_max_ values were estimated by least-square nonlinear regression analysis using GraphPadPrism 5 software. (**B**) Influence of altered extracellular pH on saracatinib accumulation into RASFs quantified by intracellular levels of saracatinib via the HPLC-UV method. Values are mean ± SEM expressed as percentage of saracatinib uptake at pH 7.4, which was set to 100%. Asterisks * indicate significant differences from saracatinib transport at pH 7.4 in RASFs, calculated by one-way ANOVA followed by Dunnett’s test (*P* < 0.05). The numbers in the columns indicate the number of experiments.

Accumulation studies in human synovial fibroblasts showed that saracatinib uptake by RASF is a saturable process, indicating a transporter-mediated mechanism with a K_m_ of 39.4 ± 9.4 µM (Fig. 2A). A similar K_m_ value of saracatinib was measured in hOCTN1-HEK293 cells. In addition, the rate of saracatinib transport capacity (V_max_) in RASF was 71.6 ± 4.1 nmol/mg protein/10 min (Fig. 2A). Accordingly, the transport efficiency (defined as V_max_/K_m_) of saracatinib uptake by RASF was 1.8 ± 0.4 ml/mg protein/10 min. Studies performed at different pH showed that saracatinib uptake in RASF is pH-dependent, further suggesting the involvement of OCTN1 in this process (Fig. 2B).

**Figure 3:**
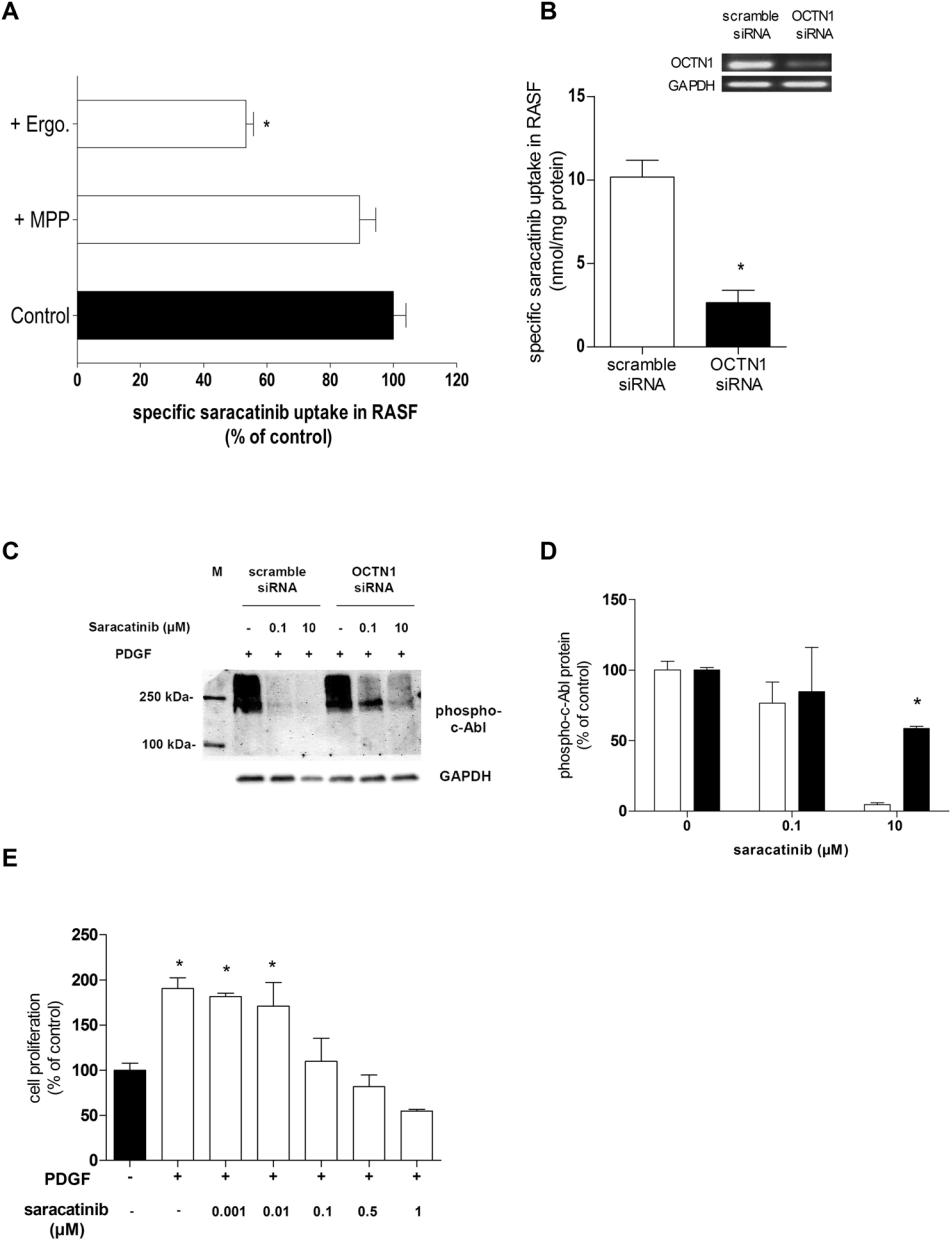
Dependence of saracatinib uptake and biological function on hOCTN1 in hRASF. (**A**) Saracatinib uptake (10 µM) by RASF under inhibition of OCT1-3 by 20 µM MPP^+^ or OCTN1 by 40 µM ergothioneine (Ergo.), respectively (n = 8–14). (**B**) Saracatinib uptake by RASF 24 h after transfection with non-targeting (scramble-siRNA, white column) or OCTN1 (OCTN1-siRNA, black column) siRNA. Data are shown as mean ± SEM of three independent experiments. (**B**, insert) Transcriptional expression levels of OCTN1 in RASF 24 h after transfection with scramble-siRNA or OCTN1-siRNA determined by PCR (a whole gel of PCR analysis for detection of siRNA-transfection effects is shown as Supplemental material Fig. 1). (**C**) Representative Western blot analysis of PDGF induced (10 ng/ml) c-Abl phosphorylation with or without (−) saracatinib incubation for 10 min in scramble-siRNA- and OCTN1-siRNA transfected RASF of one from three independent experiments. M indicates the lane with molecular weight markers. As loading control was used GAPDH, which has a lower molecular weight than phosphorylated c-Abl (phospho c-Abl). (**D**) Protein expression levels of phospho-c-Abl upon PDGF (10 ng/ml) and saracatinib incubation (0, 0.1, and 10 µM) for 10 min in scramble-siRNA- (white column) and OCTN1-siRNA (black column) transfected RASF (n = 3). Asterisks * indicate significant difference compared to scramble-siRNA-RASF incubated with 10 ng/ml PDGF and 10 µM saracatinib, calculated by two-tailed Student’s t-test for unpaired experimental values. (**E**) Effects of saracatinib on PDGF-induced proliferation of cultured RASFs. RASFs were incubated for 18 h with 10 ng/ml PDGF in the presence of the indicated saracatinib concentrations. Cell proliferation was determined by counting the number of SFs from three different RA patients. Data are shown as mean ± SEM. Asterisks * indicate significant differences compared to control with PDGF, calculated by one-way ANOVA followed by Dunnett’s test (*P* < 0.05).

To further investigate whether hOCTN1 mediates saracatinib uptake in RASF, we performed uptake competition experiments using the specific OCTN1 substrate ergothioneine^20^ and under down-regulation of hOCTN1 expression by siRNA. Ergothioneine considerably interfered with the saracatinib uptake into RASF by reducing its net accumulation rate to 53% of the control (Fig. 3A). Importantly, MPP^+^, a strong inhibitor of hOCT1-3^21^, did not influence saracatinib uptake, suggesting that hOCT1-3 did not functionally contribute to this process in RASF (Fig. 3A). To further confirm the role of hOCTN1 in this process, we performed a knockdown of hOCTN1 in RASF using specific siRNA. The suppression efficiency was confirmed by measuring the transcriptional levels of hOCTN1 using semiquantitative RT-PCR (Fig. 3B, the insert shows an example of three undependent determinations). As a result, the uptake of saracatinib was effectively decreased to 26% (2.6 ± 0.8 nmol/mg protein) in RASF 24 h post-transfection with hOCTN1 siRNA (Fig. 3B). To further address the significance of this OCTN1-mediated uptake for therapeutic effects, we investigated the impact of OCTN1 knock-down on the inhibitory effect of saracatinib on PDGF-induced c-Abl phosphorylation in RASF. As a consequence of the reduced uptake, we found that the saracatinib inhibition of the PDGF-induced c-Abl activity measured as c-Abl-phophorylation was significantly reduced in hOCTN1 siRNA-transfected cells compared to cells transfected with non targeting si RNA (scramble siRNA, Fig. 3C,D). A further hint of the potential use of saracatinib as a RA therapeutic was obtained by assessing the influence of saracatinib on PDGF-induced cell proliferation of human RASF. A potent and dose-dependent inhibition of cell proliferation was observed following 18 h saracatinib treatment within the 0.001–1 µM concentration range (Fig. 3E). Complete inhibition was evident in the presence of 0.1 µM saracatinib, a concentration known to inhibit effectively the c-Abl and Src kinases^22^. The C_max_ of saracatinib in oncologic patients treated with this TKI is between 0.1 and 1 µM, depending on the therapeutic protocol^23, 24^.

### Saracatinib transport is enhanced under pathological conditions in RASF

**Figure 4:**
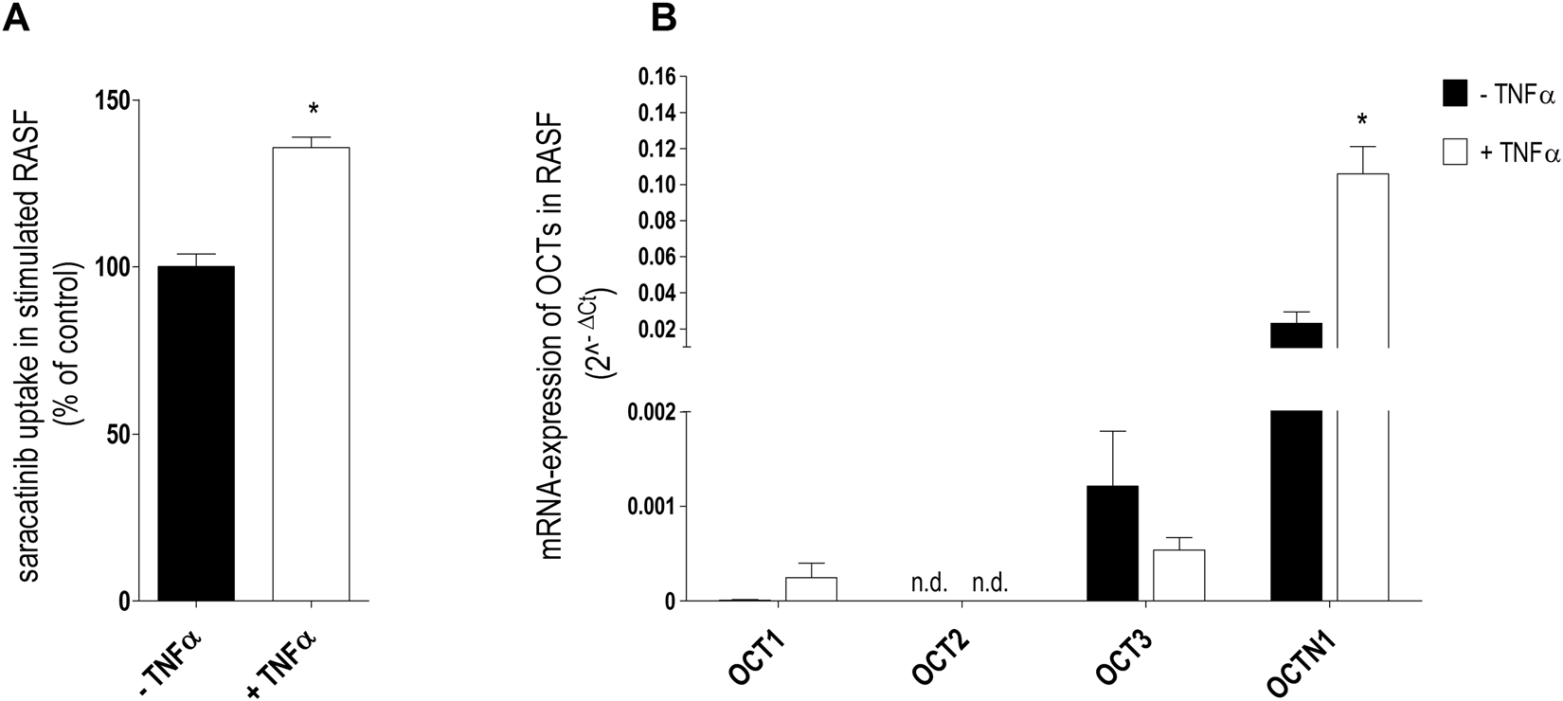
Effect of pro-inflammatory conditions on saracatinib uptake and transporter expression in hRASF. (**A**) Impact of TNFα (10 ng/ml) administration for 18 h on saracatinib accumulation in RASF (n = 15–16). Unpaired student t-test was used for statistical analysis. (**B**) Effect of 18 h TNFα (10 ng/ml) treatment on mRNA expression of investigated transporters in RASF relative to GAPDH using real-time PCR (n = 4–8). Asterisks * show statistical significant difference between neighboring columns, assessed by two-tailed Student’s t-test for unpaired experimental values. The abbreviation n.d. stands for not detected.

As the inflammatory cytokine TNFα plays a central role in SF activation and progressive joint destruction in RA^25^, its influence on the saracatinib uptake process was evaluated. We found a significant increased (36 ± 5%) saracatinib uptake in RASF treated for 18 h with TNFα (10 ng/ml) compared with untreated controls (Fig. 4A). In line with our results demonstrating hOCTN1 as the main saracatinib transport system in RASF, transcription levels of hOCTN1 were strongly up-regulated under TNFα incubation compared to the control RASF (the expression of OCTN1 was 5-fold increased, Fig. 4B). As we have also shown in previous studies^26^, hOCT1 and hOCT3 expression was not altered upon TNFα administration, suggesting that the increase of saracatinb uptake by TNFα is due to an increase of hOCTN1 expression.

## Discussion

The TKI saracatinib is a potent dual inhibitor of c-Src and c-Abl kinases, which are involved in cell proliferation, differentiation, and survival^22, 27^. Aberrant expression and activity of Src kinase have been found in RASF and have been linked to enhanced cell migration and invasion^28^. Both kinases, Src as well as c-Abl have are potential theapeutic targets in RA^6, 12–15, 17, 29^. Thus, saracatinib may be a suitable drug to antagonize the fibroblast-mediated joint erosion in RA. Since uptake of saracatinib into RASF is a prerequisite for intracellular drug action, we evaluated the uptake route of saracatinib in RASF.

Because of the cationic nature of saracatinib, we investigated whether its cellular transport is mediated by organic cation transporters and identified hOCT1, hOCT2, hOCT3, and hOCTN1 to be involved in this process (Fig. 1A). Kinetic studies revealed that hOCTN1 has a higher apparent affinity for saracatinib than the other OCT subtypes (Fig. 1B). Cellular accumulation kinetics further showed that saracatinib uptake by RASF is a saturable process, indicating a transporter-mediated mechanism. The calculated K_m_ value of hOCTN1 is within plasma concentrations reached during saracatinib therapy^23, 24^. The acidic milieu, which is a concomitant consequence of inflammatory processes, caused a decrease of saracatinib transport in RASF (Fig. 2B) owing to the fact that hOCTN1 functions as a H^+^/organic cation antiporter^30, 31^.

By competition and down-regulation experiments, hOCTN1 was identified as the main transport system for saracatinib accumulation in RASF (Fig. 3A,B) and was shown to be critical for determining the molecular effects of saracatinib in RASF: down-regulation of hOCTN1 expression ultimately reduced the intracellular saracatinib action on PDGF-induced tyrosine kinase c-Abl (Fig. 3C,D). As expected from recent data on the potential of Src and c-Abl and their inhibitors on RA, we have shown for the first time that the PDGF-induced proliferation of RASF can be completely blocked by saracatinib (Fig. 3E).

Having demonstrated the critical role of hOCTN1 in mediating effective saracatinib accumulation in RASF, the influence of RA-relevant inflammatory conditions on saracatinib transport in RASF was also investigated. Since TNFα plays a key role in the induction and perpetuation of the chronic inflammatory processes in RA^32^, we assessed its influence on the translocation process of saracatinib in RASF. Following TNFα stimulation, we found an increased hOCTN1 mRNA expression in RASF and, consequently, increased accumulation rates of saracatinib (Fig. 4A,B).

These results underline the importance of an inflammatory milieau, such as that induced by TNFα, for the uptake and efficacy of TKI in general as well as for the regulation of OCTN1 expression and activity. Maeda and colleagues^33^ identified several transcription factors including NF-κB and Sp1 that induce hOCTN1 transcription. As opposed to that, other transcription factors such as RUNX1 have been reported to suppress OCTN1 transcription^33, 34^. An emerging body of evidence demonstrates the importance of NF-κB and RUNX1 signal molecules as critical regulators in autoimmunity and inflammation^35^. Indeed, several studies indicate that hOCTN1 expression and activity is changed in inflammatory diseases such as RA^36^ and Crohn’s disease^37^.

For these reasons, it can be speculated that TNFα increases hOCTN1-mediated saracatinib transport most likely by recruiting NF-κB signaling and, thereby, enhancing hOCTN1 expression. In accordance with our results, previous studies reported that TNFα promotes hOCTN1 mRNA expression in fibroblast-like synoviocytes from individuals with RA^36^. Interestingly, recent studies have shown that compared with healthy patients, erythrocytes from RA patients have increased levels of ergothioneine, a dietary antioxidant that is imported into the cells by hOCTN1^38^. High expression of OCTN1 was also observed in hematological and immunological tissues of inflamed joints from mice with collagen-induced arthritis^36^. The physiological reason for the overexpression of hOCTN1 in inflammatory conditions might thus be a protection from oxidative stress by higher ergothioneine levels.

Although it is not surprising that the Src and c-Abl inhibitor saracatinib has anti-proliferative effects on RASF, the increased uptake in inflammatory conditions makes saracatinib a promising therapeutic option for RA. In contrast to other TKI such as imatinib, saracatinib does not face the problem of reduced intracellular uptake and availability in an inflammatory milieu that has been proposed to be responsible for poor clinical effects of imatinib despite promising *in vitro* data^26^.

In conclusion, we have shown that the membrane transporter hOCTN1 is critical for saracatinib uptake into RASFand can mediate the effectiveness of saracatinib at the site of action. Furthermore, the function and expression of hOCTN1 is regulated by disease specific factors, namely PDGF, pH, and TNFα. Therefore, we suggest that detailed information about transporter expression and function is of pivotal importance for specific targeting of orally available drugs; in this way their efficacy can be maximized while simultaneously minimizing their side effects. According to our data, saracatinib might be a promising alternative for RA treatment and should be further evaluated in this context.

A critical exploration of transporter mediated drug processing is essential for developing new anti-inflammatory strategies and a reevaluation of known drugs for repositioning may be indicated in certain cases. We conclude that RA patients may have a higher likelihood of responding to saracatinib due to its transport by hOCTN1, and that hOCTN1 is an attractive molecular uptake route for development of drugs for the treatment of RA.

## Materials and Methods

### Cell lines

HEK293 cells (CRL-1573; American Type Culture Collection, Rockville, MD) were stably transfected with hMATE1-plasmid^39^ provided by Atsushi Yonezawa (Kyoto University Hospital, Japan), and selected with 0.5 mg/ml hygromycin B (Invitrogen, San Diego, USA). hOCT1-3 stably transfected HEK293 cells were a gift of Prof. Koepsell (University of Würzburg, Germany). cDNAs of the novel organic cation transporter 1 (hOCTN1) and hOCTN2 subcloned into a doxycycline-inducible pEBTetD plasmid vector^40, 41^ (provided by Prof. Gründemann, University of Cologne, Germany) were transfected in HEK293 cells and selected with 3 mg/l puromycin (Invitrogen). Twenty-four hours before starting experiments hOCTN1 expression was induced by 1 mg/l doxycycline (Sigma-Aldrich, Steinheim, Germany). Cells were grown at standard conditions. Synovial fibroblasts (SF) were isolated from tissues of patients with rheumatoid arthritis (RA) (n = 8) obtained during joint replacement surgery after written informed consent was obtained. This procedure was approved by the Ethics Committee of the University of Münster, Germany; all methods were performed in accordance with the relevant guidelines and regulations. The manuscript does not contain information or images that could lead to identification of a study participant. RA patients met the American College of Rheumatology criteria. Isolated fibroblasts were cultured under standard conditions for a maximum of eight passages. When indicated, RASF were incubated with 10 ng/ml TNFα (PeproTech, Hamburg, Germany). Cell number was evaluated with a CASEYTT cell counter (Roche Innovatis, Reutlingen, Germany).

### Determination of apparent affinities of saracatinib for transporters of organic cations with 4-(4-(dimethylamino)styryl)-N-methylpyridinium (ASP^+^)

Apparent affinities of hOCT1, hOCT2, hOCT3, and hOCTN1 for saracatinib were determined in transfected HEK293 cells by inhibiting the uptake of the known OCT substrate ASP^+^ (1 μM) with saracatinib (10^−10^ to 10^−3^ M) as described elsewhere^42^.

### HPLC-UV detection of cellular saracatinib accumulation

For HPLC detection of cellular saracatinib accumulation cells were incubated for 10 min with 10 μM saracatinib in physiological buffered saline (PBS) at 37 °C or 4 °C in the presence or not of 20 μM 1-methyl-4-phenylpyridinium iodide (MPP^+^) or 40 μM ( + )-ergothioneine as substrates of hOCT1-3 or hOCTN1, respectively. After incubation, cells were washed with ice-cold PBS and hypoosmotic lysis was induced with 0.1% formic acid. For saracatinib quantification, a high pressure liquid chromatography (HPLC) method was used. The mobile phase consisted of (A) acetonitrile with 0.1% formic acid and (B) 0.1% formic acid and was delivered at 0.3 ml/min in a gradient program. After delivering 100% B for 3 min, a linear gradient to 100% A was applied within 7 min and maintained for 5 min. Thereafter, in order to re-equilibrate the column, a simple isocratic elution step with 100% eluent B was applied for at least 6 min. The chromatographic system consisted of an Accela 600 Pump, an automatic sampler, an UV detector set to 259 nm for detection and a degasser. Separation was carried out on an Accucore C18 column equipped with a C18 guard column. The data were acquired by ChromQuest 5.0 chromatography software.

### Quantitative real-time PCR (qRT-PCR)

RNA was isolated with the Qiagen RNeasy Midikit (Qiagen, Gilden, Germany) and Invitrogen Super Script III system was used for reverse transcription. qRT-PCR was performed using SYBR Green PCR Master Mix and the ABI PRISM 7900 Sequence Detection System (Applied Biosystems, Darmstadt, Germany) (primer pairs are reported in Table 1 of supplementary materials). Relative gene expression values were evaluated with the 2^−ΔCt^ method using GAPDH as housekeeping gene^43^.

### OCTN1 silencing

To down-regulate hOCTN1 expression, RASF were transfected with either 40 nM hOCTN1 siRNA (Sigma-Aldrich; GACAAUUUACUGUGAGUUA) or non targeting scramble siRNA (Invitrogen; nonsilencing control siRNA) using a N-TER™ Nanoparticle siRNA Transfection System^®^ (Sigma) according to the manufacturer’s instructions. The transfection efficiency was verified by PCR. The dependence of saracatinib biological impact on hOCTN1 expression in RASF was assessed at the molecular level by determination of c-Abl activity. RASF transfected with hOCTN1- or scramble-siRNA were incubated with 1 μM or 10 μM saracatinib and 10 ng/ml PDGF for 10 min, and total extracts were examined for c-Abl activation by Western blotting using anti-phospho Abl (Cell signaling, Danvers, USA; diluted 1:500) and GAPDH antibody (Sigma-Aldrich). The part of membrane containing GAPDH, as determined from molecular weight markers, was cut and the expression of GAPDH was detected as loading control. Western blots were quantified by ImageJ software (NIH, Bethesda, MD, USA).

### Chemicals

Saracatinib was purchased from LC Laboratories (Woburn, USA), ASP^+^ from Molecular Probes (Invitrogen). L-(+)-ergothioneine and MPP^+^ were obtained from Sigma. Chemicals were dissolved in PBS or HCO-﻿_3_-free Ringer-like solution containing (in mM): NaCl 145, K_2_HPO_4_ 1.6, KH_2_PO_4_ 0.4, D-glucose 5, MgCl_2_ 1, calcium gluconate 1.3, and pH adjusted to 7.4 at 37 °C.

### Statistical Analysis

Data were analyzed using GraphPad Prism, Version 5.0 (GraphPad Software, Inc., San Diego, USA) and are shown as mean ± SEM. When indicated, ANOVA with Dunnet’s posthoc test or an unpaired Student t test was applied. A *P*-value < 0.05 was considered statistically significant.

## Electronic supplementary material

Supplemental Material
