# Supplementary material for: Importance of the novel organic cation transporter 1 for tyrosine kinase inhibition by saracatinib in rheumatoid arthritis synovial fibroblasts
Source: Sci Rep. 2017 Apr 28;7:1258. doi: 10.1038/s41598-017-01438-4 (PMC5430895; doi:10.1038/s41598-017-01438-4)

Supplemental materials of the manuscript: “Importance of the novel organic cation transporter 1 for tyrosine kinase inhibition by saracatinib in rheumatoid arthritis synovial fibroblasts” by

Saliha Harrach, Bayram Edemir, Christian Schmidt-Lauber, Thomas Pap, Jessica Bertrand, Giuliano Ciarimboli

Supplemental Table 1: List of primer sequences used for quantitative PCR

Primer		Sequences (5´→ 3´)
hOCT1	Forward	CAT CAT AAT CAT GTG TGT TGG CC
	Reverse	CAA ACA AAA TGA GGG GCA AGG CTT
hOCT2	Forward	CGC CAT TCC TGG TCT ACC GGC
	Reverse	GCT TCC TCG ATG GTC TCA GGC
hOCT3	Forward	GAC AAG AGA AGC CCC CAA CCT GAT
	Reverse	CAC TAA AGG AGA GCC AAA AAT GTC
hOCTN1	Forward	GTG CTG TGT GTC CCG CTG TG
	Reverse	CGA GTC CTG AAC AGG TCC AG
hOCTN2	Forward	CAT GCA GAC AGG CTT CAG CTT C
	Reverse	ATG CAC ACT CCT AAC GTA GAG
hMATE1	Forward	AAG CTG GAG CTG GAT GCA GTC
	Reverse	CAG CAG AGG AGC AGG ACG AGC
GAPDH	Forward	CAA GCT CAT TTC CTG GTA TGA C
	Reverse	GTG TGG TGG GGG ACT GAG TGT GG

Supplemental materials of the manuscript: “Importance of the novel organic cation transporter 1 for tyrosine kinase inhibition by saracatinib in rheumatoid arthritis synovial fibroblasts” by

Saliha Harrach, Bayram Edemir, Christian Schmidt-Lauber, Thomas Pap, Jessica Bertrand, Giuliano Ciarimboli

Supplemental Figure 1: Transcriptional expression levels of hOCTN1 and hGAPDH in RASF 24 h after transfection with scramble-siRNA or OCTN1-siRNA determined by PCR. The base pair length (bp) markers with the indication of the 100 bp are also shown.

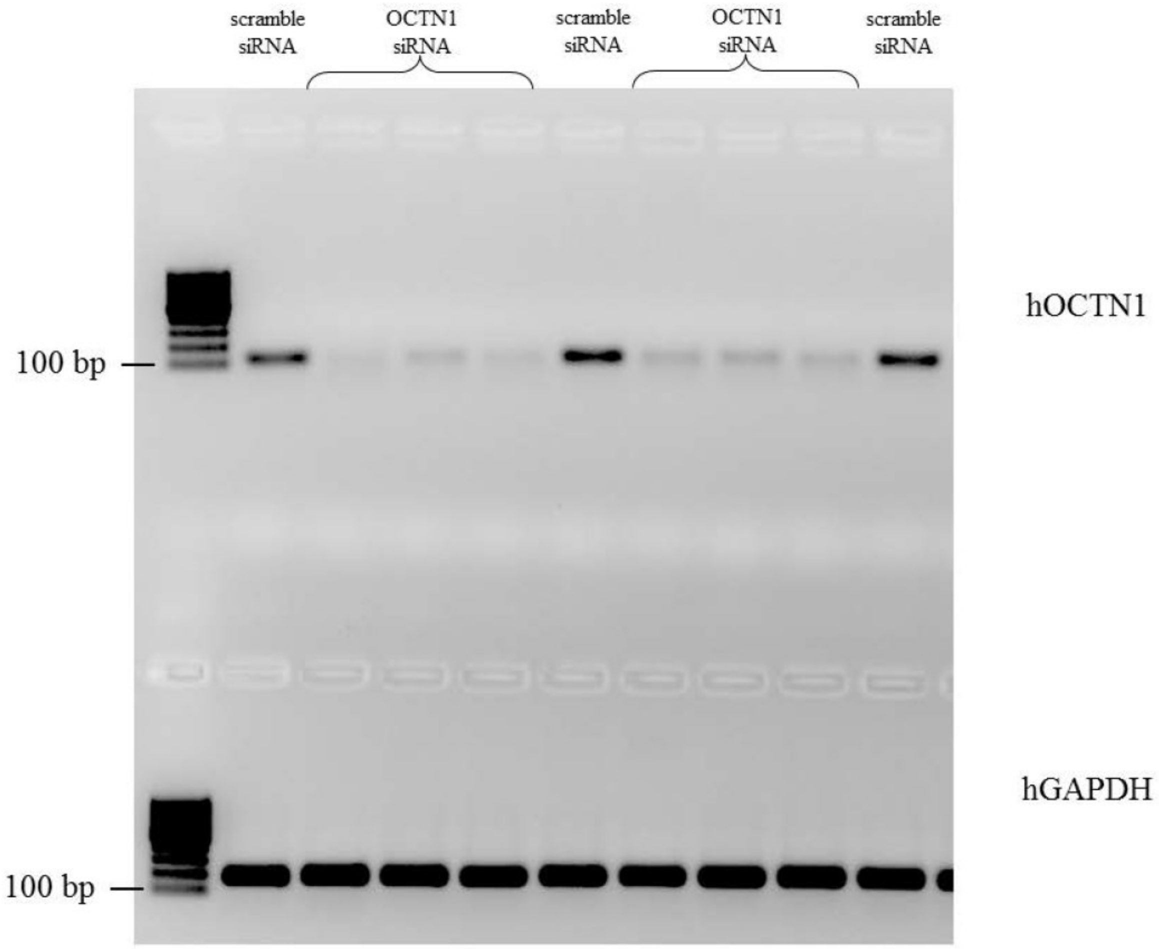

Supplemental materials of the manuscript: “Importance of the novel organic cation transporter 1 for tyrosine kinase inhibition by saracatinib in rheumatoid arthritis synovial fibroblasts” by

Saliha Harrach, Bayram Edemir, Christian Schmidt-Lauber, Thomas Pap, Jessica Bertrand, Giuliano Ciarimboli

Supplemental Figure 2: Endogenous transcriptional expression levels of transporters for organic cations in control HEK293 cells, as determined by quantitative PCR analysis. Data are shown as mean ± SEM. The number of independent experiments is indicated above the columns.

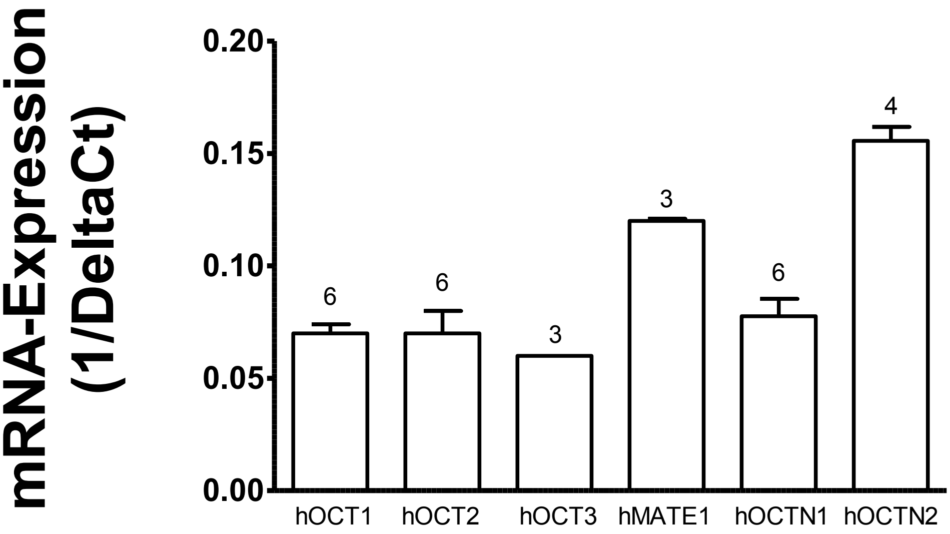

Supplement: Supplementary file 1 — Supplemental Material [file 41598_2017_1438_MOESM1_ESM.pdf]
